# Agoraphobia

**Published:** 1882-11

**Authors:** L. T. Potter

**Affiliations:** Clinical Lecturer on Diseases of the Chest, South Side Dispensary, Chicago, Ill.


### ARTICLE IV.

AGORAPHOBIA. A Contribution to Clinical Medicine. By L. T. POTTER, B.L., M.D., Clinical Lecturer on Diseases of the Chest, South Side Dispensary, Chicago, Ill.

My attention has recently been directed to an article in *The Popular Science Monthly*, by M. Bill, whom I take to be a Parisian physician of some celebrity in psychological pathology, for he certainly evidences in the subject he treats, viz.: "Delusions of Doubt," a mental erudition which amply justifies my conclusion.

In the realm of psychiatry, we are constantly discovering new and untrodden paths for investigation which demand our most careful attention and thorough research.

If, by any process of mental application, either with or without mechanical aid, we are enabled to discern in the labyrinth of cerebral pathology any new fact, we will have been more than rewarded by the finding, in that we will by just that much have increased our own knowledge and added to the literature of psychiatry; an achievement worthy of our most intent emulation.

Last winter, while reading, during my leisure evenings, "A Physician's Problems," by Chas. Elam, M.D., M.R.C.P., I was much impressed with the chapter on "Illusions and Hallucinations." The impression has been an enduring one, and I should do Dr. Elam an injustice if I did not add, that I consider him one of the most original and erudite writers with whom it has been my pleasure to enjoy an hour's literary treat after a hard day's work at the bedside practice.

But let us turn to the case which prompted this little monograph.

Last August, while taking my summer vacation, I was consulted in Providence, R. I., as to my opinion upon a case of peculiar and somewhat rare mental disease. Was it an example of so-called "conscious insanity?"

The history of the case is as follows:

Mrs. D., æt forty; brunette; nervous temperament; has had but one child. Is unable to state just when she first noticed symptoms of her disease. When taken into a railway car, so intense was the agony of her situation after the car was in motion, it seemed as though she would die. Repeatedly had she attempted journeying by rail, only to be compelled to get off and ride by carriage the remaining distance

The sensations experienced she described to be, when the car was moving, as though she was being swung from a precipice. If her attention is attracted by some one speaking to her, the sensation leaves her in a moment; only, however to return again in another moment. She gasps one moment and laughs the next. Is always much worse a week before her menstrual period.

For years she has been obliged to forego the pleasure of traveling by rail, and resort to conveyance by horse, when wishing to visit a point inaccessible by water. Never experiences any difficulty when traveling by water. The sensations referred to are produced only when in a moving railroad car.

She had observed that some individuals seemed capable of exerting a quieting influence over her on these occasions, while other persons had the opposite effect; an illustration of psychological control; the stronger intellect controlling the weaker.

Loud music in church or concert halls was unbearable to her. She remembers, as a little child, when exposed to a current of air, to have experienced symptoms of a nervous character, which I should judge simulated chorea. As she approached puberty, her symptoms improved, but the improvement was not of long duration.

After the death of a favorite cousin, her nervous symptoms increased; appetite became impaired; depressed in spirits; was wakeful at night, and if perchance she did fall asleep, would awaken tired and unrefreshed. She has noticed that if interested in something which required close mental application, she does not feel the morbid sensations.

Having consulted many physicians in Providence, and taken much internal medication, with no alleviation of her sufferings, she at length came under the care of Dr. G., a prominent surgeon, also of Providence. He seemed much interested in her case, and after having made a most careful and painstaking examination, concluded that some of her symptoms, if not all, were due to an abrasion or laceration of the mucous membrane of the os uteri. With this conception of the etiology of her trouble, he made local applications to the cervix and os, and the patient's symptoms steadily improved. This was about two years ago; since which time she has been able to sleep and eat very well.

Still, even at the time I saw her, she was unable to travel without some trepidation. A bottle of valerianate of ammonia, and a flask well filled with brandy, were always her constant companions when undertaking a journey by rail.

An amusing side of the case lies in the fact that when she is traveling, she invariably sits with a brandy flask in the right hand, and her Bible in the left; presumably the one counteracting the influence of the other.

The interesting question which suggests itself to every thinking mind, when brought in contact with a rare neurotic case like this is: "What is the cause?" Having once clearly determined that, the success of our subsequent treatment is assured.

Did the last physician this lady employed find the correct solution of a disease which had given rise to years of suffering, both corporeal and psychological, to the patient, and baffled the skill of her former medical advisers?

I am strongly inclined to believe that the lesion of the cervix uteri at the os was, if not the origin, in a great measure an aggravating factor, which, until relieved, prevented a complete recovery. Does not the very fact that she improved so steadily by the use of local applications, with internal antispasmodics, go far to favor this theory? It appears to me it does, beyond any peradventure or doubt.

Just as trifling a cause has been known to give rise to a *grand-mal*. All of us have known of instances where an adherent prepuce has given rise to convulsions, epilepsy, etc. The constant irritation at the periphery of a nerve filiament has been recognized as the cause of tetanic spasm. Cases might be multiplied as exemplifying this theory.

But now, if I am asked the more difficult question, *how* it comes to pass that such apparently insignificant lesions are capable of producing such serious effects, I must confess my ignorance. I willingly refer you to some recondite neurologist, to whom the answer is probably as clear as the differentiation between pleuritis and endocarditis is to you or me.

There is a lesson of wide application to be learned from the history of a case like this.

Can we search too carefully for the cause in any obscure disease? There is no truer aphorism than this: "The success of the medical practitioner depends upon a thorough knowledge of the *little* things in the practice." It is also none the less true that the success is all the greater when achieved through the medium of a brilliant diagnosis. He who, by carefully weighing and digesting the facts attainable in any given obscure case, is enabled by his closer mental application and acumen to discern correctly and intelligently the etiology of the aforesaid complaint, and then rectify it, deserves as much, yes, more honor and praise from those of us engaged in the active practice of medicine, than is, unfortunately, too readily bestowed upon some surgeon who has operated with the knife, possibly far more skilfully, but with much less intellectual erudition.

